# Kabuki and CHARGE syndromes: overlapping symptoms and diagnostic challenges

**DOI:** 10.31744/einstein_journal/2025RC1142

**Published:** 2025-02-07

**Authors:** Bruno Pellozo Cerqueira, Elenice Andrade Milhomem, Ana Cristina Carvalho de Matos, Igor Gouveia Pietrobom, Carlos Magno Leprevost, Ita Pfeferman Heilberg

**Affiliations:** 1 Universidade Federal de São Paulo Escola Paulista de Medicina Nephrology Division São Paulo SP Brazil Nephrology Division, Escola Paulista de Medicina, Universidade Federal de São Paulo, São Paulo, SP, Brazil.; 2 Secretaria de Saúde do Paraná Paraná PR Brazil Secretaria de Saúde do Paraná, Paraná, PR, Brazil.

**Keywords:** Kabuki syndrome, CHARGE syndrome, KMT2D, Hypoparathyroidism, Genetic testing, Diagnosis, differential, Choanal atresia

## Abstract

Kabuki syndrome is a rare congenital malformation with typical facial features, skeletal anomalies, delayed neuropsychomotor development and growth, and cardiac, genitourinary, gastrointestinal, endocrine, and dental anomalies. One of the main differential diagnoses is CHARGE syndrome, standing for and characterized by Coloboma of the eye, Heart defects, Atresia of the nasal choanae, Restricted intellectual development, Genitourinary malformations, and Ear anomalies. Because these syndromes have similar characteristics, distinguishing them may be challenging. A 24-year-old male patient admitted with reduced renal function had a previous phenotype-based diagnosis of CHARGE syndrome based on many characteristic clinical features. The unveiling of a hypocalcemic crisis diagnosed as primary hypoparathyroidism at the age of 15 years, which did not fit into that diagnosis, led the nephrologist to request a genetic test, which evidenced a missense variant of uncertain significance in exon 38 of the *KMT2D* gene. This phenotype further suggested Kabuki syndrome, ruling out CHARGE. The present report highlights the importance of genetic testing and discusses phenotype-genotype correlations, which ultimately showed that specific variants in exon 38 rendered a form of Kabuki syndrome distinct from the typical one.

## INTRODUCTION

Kabuki syndrome (KS) is a rare autosomal dominant condition that arises *de novo* in the majority of cases. It was first described in 1981 in Japan, with an estimated prevalence of 1:32,000 live births.^(1)^ In 2004, a study estimated a minimum birth prevalence of 1:86,000 in Australia and New Zealand.^(2)^ KS is characterized by typical facial features, skeletal anomalies, delayed neuro-psychomotor development and growth, and cardiac, genitourinary, gastrointestinal, endocrine, and dental anomalies. Typical facial findings include long palpebral fissures and eversion of the lower eyelids. The disorder was originally named Kabuki-makeup syndrome, because the facial features of many affected children resembled the makeup used by actors in kabuki, a form of Japanese theater. Disease-causing variants associated with KS occur in the *KMT2D* gene encoding KS-1, a protein with a methyltransferase function, in 75% of cases^(3)^ or in *KMD6A*, encoding KS-2 with a demethylase function, in 5% of cases, which follow an X-linked dominant inheritance pattern. In 20% of cases, the etiology is unknown.^(3,4)^ Both genes participate in embryogenesis and development.^(5)^ A recent international consensus reviewed the criteria for KS diagnosis,^(6)^ and a phenotypic score was suggested by other investigators to help predict genotype variants in the *KMT2D* gene in patients with clinical suspicion of KS.^(7)^

One of the main differential diagnoses of KS is CHARGE syndrome, standing for and characterized by Coloboma of the eye, Heart defects, Atresia of the nasal choanae, Restricted intellectual development, Genitourinary malformations, and Ear anomalies. The diagnostic criteria were described by Blake et al.^(8)^ and Verloes.^(9)^ This syndrome is a rare, usually sporadic, autosomal dominant disorder with an approximate incidence of 1:10,000 live births and is caused by pathogenic variants in the *CHD7* gene in 2/3 of cases (chromodomain helicase DNA-binding protein).^(10,11)^ Approximately 90% of patients have a heterozygous variant of this gene, mostly *de novo* and in an autosomal dominant pattern.^(10)^

Given the similarities between the phenotypes, their differentiation has become challenging.^(12–14)^ This study identified a missense variant in exon 38 of the *KMT2D* gene in a patient with a history of congenital anomalies and a phenotypic diagnosis of CHARGE syndrome. The aim of this report was to discuss the diagnostic difficulties of distinguishing CHARGE from KS, expand the clinical phenotype of KS, and highlight the growing importance of genetic testing in the diagnosis of rare diseases.

## CASE REPORT

A 24-year-old male with reduced renal function was referred to a nephrologist. He was born prematurely with congenital hypothyroidism, atrophic nipples, low-set ears, hypospadia, choanal atresia, and a surgically corrected branchial fistula. The patient was phenotypically diagnosed with CHARGE syndrome. Recurrent urinary infections followed by bilateral vesicoureteral reflux and hypospadias were surgically corrected at the age of 2 years. He also exhibited abnormal dentition, with small front teeth and triangular canines, requiring dental implants, and severe sensorineural hearing loss, using hearing aids since the age of 3. At 15 years of age, he presented to the emergency room with general body pain and was diagnosed with hypocalcemic crisis. His laboratory workup suggested the presence of primary hypoparathyroidism: ionized calcium, 0.69 mmol/L (reference 1.20-1.40); PTH, 20.3 pg/mL (reference 20-65); vitamin D, 6.1 ng/mL (reference >30); and phosphate, 7.7 mg/dL (reference 2.5-4.5). Calcium and vitamin D supplementation was initiated. During the same period, due to education difficulties since elementary school, the patient underwent a neuropsychological assessment that identified a mild intellectual disability. At the age of 18, he had a glomerular filtration rate of 76.2 mL/min/1.73 m² estimated via the CKD-EPI formula (2021). Upon his admission for nephrological consultation at 24 years old, the creatinine clearance was 59.2 mL/min/1.73 m², and a DMSA renal scintigraphy showed reduced right kidney function (25%). Up to this point, no *CHD7* gene sequencing had been ordered by any physician.

No pathogenic variants were obtained using a next-generation sequencing (NGS) panel related to congenital kidney and urinary tract (CAKUT) anomalies including *CHD7*. In addition, clinical hypoparathyroidism is not a common finding in CHARGE syndrome. Thus, a second round of gene studies was required based on the dysmorphological features, including FGFR1 - Kallman syndrome*, KMT2D -* KS*, KMD6A -* KS*, RERE -* neurodevelopmental disorder with or without anomalies of the brain, eye, or heart*, SOX2 -* SOX2 disorder*, TCOF1 -* Treacher Collins syndrome, and *ZEB2 -* Mowat-Wilson syndrome*.* Genetic testing revealed a heterozygous variant of uncertain significance (VUS) (dbSNP) in exon 38 of the *KMT2D* gene [NM_003482.4:c.10595 T>C; p (Ile3532Thr)]. Considering this phenotype, KS was suspected. The variant was not identified in the parents, classifying it as *de novo*. After reviewing the variant with an enhanced phenotype and the new molecular information, it was then reclassified as likely pathogenic (LP), allowing the final diagnosis of KS.

ClinVar^(15)^ and Varsome^(16)^ included only four submissions of this variant between 2019 and 2021, including the one from the present case report. The authors disagree on the pathogenicity of this variant, with one VUS, two LP, and one pathogenic classification. The genotype-phenotype correlation disclosed in the present study informed the genetic testing provider to justify the reclassification of the variant from VUS to LP, according to the criteria described by Lincoln et al.^(17)^ as follows: 1) This sequence alteration replaces isoleucine with threonine at codon 3,532 of the KMT2D protein (p.Ile3532Thr). The isoleucine residue is moderately conserved, and isoleucine and threonine exhibit a moderate physicochemical difference. 2) This variant is not present in population databases (ExAC, no frequency). 3) This missense change has been observed in individuals with KMT2D-related diseases. 4) The variant is observed *de novo*. 5) Algorithms developed to predict the effect of missense changes on protein structure and function are unavailable or do not agree on the potential impact of this missense change. In conclusion, the variant showed strong indications of being pathogenic, but additional data, such as functional tests, are needed to confirm its pathogenicity. This study was approved by the Research Ethics Committee of *Universidade Federal de São Paulo* (CAAE: 76488723.1.0000.5505, # 6.722.535).

## DISCUSSION

This case shows the importance of genetic testing to confirm diagnoses, as the patient was referred to a nephrologist with a clinical diagnosis of CHARGE syndrome; however, genetic analysis of the *CHD7* gene was negative. Table 1 shows different criteria for the clinical diagnosis of both syndromes, with some overlap. According to the Blake et al.^(8)^ criteria (the "four C's"), the patient only met two major criteria (choanal atresia and characteristic ear abnormalities) and one minor criterion (development delay). According to the Verloes criteria,^(9)^ the patient exhibited atypical CHARGE syndrome with one major (choanal atresia) and two minor criteria (ear abnormalities and mental disability). When we evaluated the phenotypic score proposed by Makrythanasis et al.^(7)^ for KS, the score was low (2 points) and marked by up to three facial features (1 point) and renal manifestations (1 point). This score is only used to compare KS phenotypes and has no diagnostic role. In the study that led to the scoring system, patients diagnosed with KS and a pathogenic variant of the *KMT2D* gene had an average score of 6.1, compared to 4.5 in those without the variant. According to the criteria established by the international consensus,^(6)^ the patient only met the definitive diagnosis of KS after NGS testing and refinement of the *KMT2D* variant classification associated with a history of hypotonia and mild intellectual deficit.

**Table 1:** Main diagnostic criteria for CHARGE and Kabuki syndrome in the literature

CHARGE syndrome		Kabuki syndrome	
Blake et al. criteria^(6)^	Verloes criteria^(7)^	Phenotypic scoring system^(5)^	International consensus^(4)^
Major criteria		Facial features (0-5 points)^†^	Major criteria
Coloboma	Coloboma	Eyes: arched eyebrows, sparse lateralone third; blue sclera; everted lower eyelids; long palpebral fissures; ptosis; strabismus;	A pathogenic or likely pathogenic variant in *KMT2D* or *KMD6A*
Choanal atresia/stenosis	Choanal atresia/stenosis	Ear: large dysplastic ears	Typical dysmorphic features at some point in life: long palpebral fissures with eversion of the lateral third of the lower eyelid and two or more of the following:
Characteristic ear abnormalities	Hypoplastic/aplastic semi-circular canals	Teeth: abnormal dentition; oligodontia	Eyes: arched and broad eyebrows, with the lateral third displaying notching or sparseness
Cranial nerve involvement		Nose: broad nasal root; flat nasal tip; high/cleft palate	Nose: short columella with depressed nasal tip
		Jaw: micrognathia	Ear: large, prominent, or cupped ears
		Lips: lip nodules; thin upper and full lower lip	Finger: persistent fingertip pads
**Minor criteria**		**Limb/extremity features (up to 1 point)** ^† †^	**Supportive clinical features**
Cardiovascular malformations	Abnormal middle or external ear	Brachydactyly or clinodactyly	Constitucional: short stature
Development delay	Hypothalamo-hypophyseal dysfunction	Hip dislocation	Cardiac: congenital heart defects
Distinctive facial features	Malformation of mediastinal organs (heart, esophagus)	Lax joints	Craniofacial: microcephaly, cleft palate, lip pits, oligodontia and/or abnormal incisors, progressive sensorineural hearing loss
Genital hypoplasia	Mental disabibility	Persistent fingertip pads	Endocrinological: hyperinsulinaemic hypoglycaemia in infancy
Growth deficiency	Rhombencephalic dysfunction	**Other features**	Gastrointestinal: feeding difficulties
Orofacial cleft		Heart (1 point); kidney (1 point), microcephaly (1 point), short stature (1 point)	Genitourinary: malpositioned kidneys, hypospasdia in males
Tracheoesophageal fistula			Immunological: hypogammaglobulinaemia or low serum IgA, idiopathic thrombocytopaenia purpura
			Musculoskleteal: brachydactyly, non-traumatic joint dislocation
**Diagnosis**		**Classification**	**Diagnosis**
Definitive: four major or three major and three minor	Typical: three major or two major and two minor	^†^0-3 features = 1 point; 4-6 = 2 points;7-9 = 3 points; 10-12 = 4 points; 13-15 = 5 points	Definitive: patient of any age with a history of infantile hypotonia, developmental delay and/or intellectual disability, and one or both major criteria
Probable: three major and many minors	Partial: two major and one minor	^††^0-1 features = 0 points; 2-4 = 1 point	Probable: history of infantile hypotonia, developmental delay and/ or intellectual disability, and long palpebral fissures with eversion of the lateral third of the lower eyelid and at least three supportive features
	Atypical: one major and two minor	Phenotypic scoring system to predict which individuals with features of KS were more likely to have a heterozygous pathogenic variant in *KMT2D*. Individuals with heterozygous pathogenic *KMT2D* variants had a statistically significantly higher mean score (6.1) than those without an identified pathogenic variant in *KMT2D*^(4.5)^	Possible: history of developmental delay and/or intellectual disability and at least two supportive features and at least one dysmorfic features: arched and broad eyebrows with the lateral third displaying notching or sparseness; short columella with depressed nasal tip; large, prominent, or cupped ears; persistent fingertip pads

KS: Kabuki syndrome.

Choanal atresia is one of the major criteria for CHARGE syndrome and is present in up to 71% of cases;^(10)^ however, it is a rare finding in KS with typical features. Most variants of the *KMT2D* gene that cause KS are *de novo* and nonsense, leading to loss of function, whereas missense variants are observed in approximately 15-20% of cases. Choanal atresia is rare even in cases with nonsense pathogenic variants.^(18)^ However, data have shown missense mutations in exons 38 and 39 that cause malformations other than those described in the classic KS diagnostic criteria.^(18–21)^ Comparing the phenotypic features observed in the present patient with the ones pooled from 21 patients with missense mutations in exon 38^(18–21)^ identified the following prevalences: hearing loss (90%), choanal atresia (71%), hypothyroidism (52%), dental anomaly (48%), atrophic nipple (43%), external ear abnormalities (43%), hypoparathyroidism (19%), and kidney disease (10%). However, many other clinical features were not observed in the current case, such as abnormalities of the lacrimal ducts (57%), short stature (38%), and feeding difficulties (29%). Balbridge et al.^(20)^ reported four cases of KS that included additional features, such as choanal atresia and hypoparathyroidism, as observed in the present case, suggesting an expansion of the KS-1 phenotype. Interestingly, similar to the present case, these investigators^(20)^ found all variants to be missense and *de novo* in their series. Our variant (p.Ile3532Thr) was located in the terminal region of the *KMT2D* gene between p.(Leu3525Pro) and p.(Leu3564Val), as described previously, possibly resulting in the same range of protein alteration locations. Online Mendelian Inheritance in Man (OMIM)^(22)^ describes variants in the *KMT2D* gene in association with two distinct phenotypes, KS-1 (147920) and (620186) Branchial arch abnormalities, Choanal atresia, Athelia, Hearing loss, and Hypothyroidism (BCAHH) syndrome. Similar to others,^(20)^ we consider the latter as an expanded phenotypic spectrum of features seen in KS, rather than as a new disease. Notably, the BCAHH denomination related to the same gene and locus (last reviewed at OMIM in April 2023) has not been associated with any publication in the PubMed database to date. Table 2 shows the clinical data available for CHARGE syndrome and KS, including new characteristics.

**Table 2:** Main clinical data of CHARGE and Kabuki syndromes

	CHARGE syndrome	Kabuki syndrome
	Genetic information	
Omim number	#214800	#147920 / #300867
Gene and inheritance	*CHD7* (AD)	*KMT2D* (AD) / *KMD6A* (XL)
Chromosome location	8q12.2	12q13.12 / Xp11.3
**Main clinical findings**		
Dysmorphology features	Coloboma	Long palpebral fissures
	Cleft lip/palate	Everted eyelids
	Characteristic ear abnorbilities	Cleft lip/palate
	Choanal atresia	Ear abnorbilities
		Choanal atresia‡
		Abnormality of lacrimal ducts‡
		Branchial fistula‡
Neurological	Intellectual disability	Intellectual disability
Endocrine	Hypothyroidism	Hypothyroidism
	Parathyroid hypoplasia	Hypoparathyroidism‡
		Premature thelarche
Cardiovascular	Usually conotruncal, AV canal and aortic arch abnormalities	Most common coarctation of the aorta and septal defect
Genitourinary	Kidney and ureteral disease	Kidney and ureteral disease
	Genitalia abnormalities	Genitalia abnormalities
Growth	Postnatal growth retardation	Postnatal growth retardation

‡ Symptoms not described in classic Kabuki syndrome (KS-1), but extended KS-phenotypes^(18-21)^ or as branchial arch abnormalities, choanal atresia, athelia, hearing loss, and hypothyroidism syndrome.

AD: autosomal dominant; XL: X-linked; AV: atrioventricular.

Taken together, these clinical signs and symptoms have been described in the last decade, but they were unknown by the time the patient was born. Previously, the presence of choanal atresia led to the first phenotypic diagnosis of CHARGE, which should have been confirmed by sequencing of the *CHD7* gene. Interestingly, Schulz et al.^(14)^ demonstrated that *CHD7* and *KMT2D* interact with the WAR complex (WDR5, ASH2L, and RbBP5 proteins), a stimulator of histone H3 lysine 4 (H3K4) methyltransferase activity that participates in gene transcription, which may explain these overlapping characteristics. *KMT2D*, identified and characterized in 1997, encodes an H3K4-specific methyltransferase essential for H3K4 di- and tri-methylation, which are markers of active transcription. The KMT2D complex promotes the transcription of constitutively expressed genes via H3K4 trimethylation. As part of the ASC-2 complex (ASCOM), KMT2D facilitates binding to target genes. ASCOM actions include removing repressive epigenetic marks, displacing polycombs, and positioning activating methylation marks.^(23)^

This report presents a challenging case due to the overlapping phenotype between CHARGE and KS associated with a *de novo* missense variant in exon 38 of the *KMT2D* gene. In addition, recent data have highlighted characteristics that differ from the typical presentation of KS in patients with missense variants in this specific location, expanding its phenotype and increasing the significance of genetic testing, as well as functional and molecular studies in rare diseases.
